# Uropathogenic bacterial profile and antibiotic susceptibility pattern of isolates among gynecological cases admitted to Jimma Medical Center, South West Ethiopia

**DOI:** 10.1038/s41598-023-34048-4

**Published:** 2023-05-01

**Authors:** Sisay Teferi, Zewdineh Sahlemariam, Mekidim Mekonnen, Rahel Tamrat, Teshome Bekana, Yonas Adisu, Tefera Darge

**Affiliations:** 1 Department of Medical Laboratory Science, College of Health Sciences, Mettu University, Metu, Ethiopia; 2 School of Medical Laboratory Science, College of Medical Sciences, Institute of Health, Jimma University, Jimma, Ethiopia; 3 Department of Medical Laboratory, College of Medical Science, Wollega University, Nekemte, Ethiopia; 4 Department of Biomedical Science, College of Health Sciences, Mettu University, Metu, Ethiopia

**Keywords:** Microbiology, Medical research

## Abstract

Urinary tract infection (UTI) is one of the most common bacterial infections in women; about 50% of women get during their life time. Moreover, it is a common health problem in patients with gynecological pathologies, which increases the chance of acquiring infection. The aim of this study was to determine the bacterial profile that causes UTI and their antibiotic susceptibility pattern among admitted gynecological cases. A cross-sectional study was conducted in south west Ethiopia region. A total of 386 patients admitted with gynecological cases were recruited by sequential sampling technique and structured questionnaire was used to collect socio-demographic and risk factor-related data. About 10 ml freshly voided midstream and catheterized urine specimens were collected using sterile containers. Identification of isolate was done using culture characteristics, gram staining, and a series of biochemical tests. The antibiotic susceptibility test was performed as per the Kirby–Bauer disc diffusion technique. The data obtained were entered into EpiData Version 3.1 and analyzed using SPSS Version 25. A *P* value of less than 0.05 was used as a level of significance. In this study, the overall prevalence of UTI was 25.4%. *Escherichia coli* was the most frequently isolated bacteria, which accounted for 38 (37.6%), followed by *Klebsiella* species 22 (21.8%), CONS 14 (13.9%), *Staphylococcus aureus* 10 (9.9%), *Enterobacter* species 6 (5.9%), *Citrobacter* species 5 (4.9%), *Proteus mirabilis* 4 (4%), and *Pseudomonas* aeroginosa 2(2%). Histories of UTI (AOR = 1.977, 95% CI 1.06, 3.68, *P* = 0.032) and catheterization (AOR = 2.38, 95% CI 1.28, 4.45, *P* = 0.006) were found to be statistically associated with significant bacteriuria. Gram-negative isolates showed a high level of resistance, 88.3% for ampicillin and 66.2% for tetracycline, and a relatively low level of resistance against ceftazidime, 22.1%, and meropenem, 3.9%. Gram-positive uropathogens showed a high level of resistance to penicillin, 91.6%, whereas all isolates were sensitive 100.0% to nitrofurantoin*.* Furthermore, 80 (79.2%) of the isolates had multidrug resistance, and 16 (26.7%) of both *E. coli* and *Klebsiella* spp. produced Extended spectrum *β*-lactamase (ESBL). In this study, a high prevalence of uropathogenic bacteria and multidrug resistance for commonly prescribed drugs were observed with a significant number of ESBL producers. Therefore, screening admitted gynecological patients, especially for those who have history of catheterization and UTI, by urine culture and antimicrobial susceptibility testing is important.

## Introduction

Urinary tract infection (UTI) is the most frequent human-acquired bacterial infection which affects about 150 million people all over the world each year^1^. It involves anywhere in the urinary tract, including the kidney, ureter, bladder, and urethra. It is one of the most common bacterial infections in women^2^, more than 50% of women get during their life time^3^. This can be due to the ascent of a number of organisms into the bladder is easier than in men because of the relatively short urethra, the absence of bactericidal prostatic secretion, and the ease of contamination of the urinary tract with fecal flora^4^.

In patients with gynecological problems, blockage of urine flow^5^, anatomical and physiological changes^6^, incomplete bladder emptying, frequent bladder infections, incontinence of urine and stool, and catheterization after surgical procedures may further predispose them to urinary tract infections^7,8^. During and after gynecological surgery, bladder drainage by transurethral foleys catheter is common practice used to monitor urine output and prevent post-operative urinary retention, which is associated with an increased risk of UTIs in patients, and the daily risk of acquisition of bacteriuria when an indwelling catheter is in-situ is 3–7%^9,10^. It is known that, UTI poses a high risk of morbidity, mortality, and significant health care costs^11^. Moreover, among patients with gynecological problem, it has been a matter of morbidity, anxiety, and long hospital stays, which occurs more frequently during the postoperative period^12^.

In developing countries including Ethiopia, management of UTIs is usually empirical which may contributes for the emergency and spread of antimicrobial resistant strain which is a leading cause of treatment failure in UTI^13^. As a result, clinicians are left with very limited drug choices for the treatment of urinary tract infections. One of the leading antimicrobial resistance mechanisms for many UTI causing Gram-negative bacteria is extended-spectrum β-lactamase enzyme production that hydrolyzes the β-lactam ring of antimicrobials, which gives bacterial resistance to commonly prescribed antibiotics including penicillins; first, second and third-generation cephalosporins, aztreonams^14^.

Generally, most admitted gynecological cases need prolonged hospitalization and more intensive nursing care like prolonged bladder catheterization, which may contribute to the development of urinary tract infections that require extensive treatment with antibiotics. Therefore, this study was conducted to determine the bacterial etiologic agents of uropathogens and evaluate their in vitro susceptibility pattern to commonly used antibiotics among admitted gynecological cases at tertiary hospital in southwest Ethiopia.

## Methods

### Study design, setting, and population

A cross-sectional study was conducted among gynecological cases admitted from September 16 to December 30, 2021 at Jimma University Medical Center, South West Ethiopia. The study area is located 348 km away from the capital city, Addis Ababa. The annual average admission of the centre is over 20,000 patients. The service delivery sites are the outpatient department, gynecology ward, maternity and labor ward, and operation rooms. All gynecological cases admitted to the gynecology ward during the study period were included in the study population, and those treated with antibiotics within 15 days of the study's start date were excluded.

### Sample size and sampling techniques

The sample size was calculated using a single population proportion formula $n = \frac{{\left( {Z^{\frac{a}{2}} } \right)P\left( {1 - P} \right)}}{{d^{2} }}$, where n = number of sample size, Z is the statistics corresponding to a 95% level of confidence (1.96), d = margin of error, and P = is the assumed prevalence of uropathogenic bacteria among gynecologic cases (50%). The infection prevalence was assumed to be 50% because the current status of uropathogenic bacterial infection in the area is unknown. Therefore, the sample size was adjusted to 384 gynecological cases. A sequential sampling method was used to find study participants.

### Data collection methods

Information on demographic variables was collected from each patients with gynecolological cases by a face-to-face interview using a structured questionnaire. Clinical data was gathered through a review of patients' medical records and consultation with a gynecologist.

#### Specimen collection

After obtaining informed consent or assent from study subjects and/or parental/guardian, about 5–10 ml of urine was collected from patients by following aseptic technique and clean catch mid-stream urine (MSU) using a sterile screw-capped, wide-necked container. For those patients on a catheter, the sample was collected by aseptic techniques: cleansing the catheter port with alcohol and allowing drying time, and then aspirating the urine from the indwelling catheter with a sterile syringe. The container was labeled with the date, the name, and a code number. After collection, the specimen was immediately delivered to the microbiology laboratory for laboratory investigation. The specimens were processed within 2 h of collection. In the event of a delay in processing, the urine specimens were refrigerated at 4 °C until they were processed.

#### Culture and identification techniques

The collected urine sample was inoculated onto 5% Blood Agar, Mannitol Salt agar and MacConkey agar plates (Oxoid Ltd., Bashingstore Hampaire, UK) by streak plate methods following the standard microbiological techniques and procedures^15^. After incubating the plates aerobically at 37 °C for 24 h, they were inspected for the presence or absence of bacterial growth. If colonies were found, they were counted and multiplied by the reciprocal of the loop’s volume, or 1000. Counting colonies yielding bacterial growth of ≥ 10^5^ cfu/ml with pure growth were considered as significant bacteriuria, and negative cultures contained no growth or mixed urogenital flora (more than two isolates). For cultures containing two types of colonies, sub-culture for further identification and antimicrobial susceptibility testing. For catheter collected urine samples, the presence of symptoms or signs compatible with UTI with no other identified source of infection along with ≥ 10^3^ colony-forming units/ml and the presence of ≥ 10^5^ cfu/ml in a single catheter urine specimen in a patient without symptoms compatible with UTI is recommended as representing significant bacteriuria^16,17^.

All positive urine cultures showing significant bacteriuria were further identified by their physical characteristics such as colony morphology, odor, swarming, and presence of hemolysis on their respective media, Gram-reaction, and pattern of biochemical reactions using the standard procedures^15^. The gram-negative rods were identified with the help of a series of biochemical tests, namely Citrate, Oxidase, Sulphur Indole Motility (SIM) media, Kligler’s Iron Agar (KIA), lysine decarboxylase, lactose fermentation, urea hydrolysis^18^*.* Gram-positive cocci were identified based on their Gram reaction, mannitol fermentation, catalase and coagulase tests^15^.

### Antimicrobial susceptibility testing (AST)

The Kirby–Bauer disc diffusion method was employed for antibiotic susceptibility testing as recommended by Clinical Laboratory Standards Institute (CLSI) 2020 guidelines^19^. When a pure culture with significant bacteriuria was obtained, a loopful of bacteria was taken from a colony and transferred into a tube containing 5 ml of sterile normal saline (0.85% NaCl) and mixed gently until it formed a homogenous suspension. The turbidity of the suspension was adjusted to an optical density equivalent to 0.5 McFarland standards. The inoculated plates were left at room temperature to dry for 3–5 min while the Petridish lids were in place.

The following antimicrobial discs with their respective concentration were used for susceptibility testing and all the antimicrobials used for the study were obtained from Oxoid Ltd in the following concentrations: ciprofloxacin (CIP, 5 µg), trimethoprim-sulfamethoxazole (SXT, 1.25/23.75 µg), nalidixic acid (NA, 30 μg), meropenem (MEM, 10 μg ), cefotaxime (CTX, 30 µg), ceftazidime (CAZ, 30 µg), tetracycline (TTC, 30 µg) nitrofurantoin (F, 30 µg), norfloxacin (NOR, 10 µg), ceftriaxone (CRO, 30 µg), amoxicillin-clavulanic acid (AMC, 20/10 µg), ampicillin (AMP, 10 µg), gentamicin (CN, 10 µg), erythromycin (E, 15 µg), penicillin (PEN, 10 µg), clindamycin (DA, 10 µg) and chloramphenicol (C, 30 µg). Among these, nalidixic acid (NA), meropenem (MEM, 30 μg), cefotaxime (CTX, 30 µg), ceftazidime (CAZ, 30 µg), tetracycline (TTC, 30 µg) can be used only for gram-negative bacteria, whereas erythromycin (E, 15 µg), penicillin (PEN, 10 µg) and clindamycin (DA, 10 µg) can be used only for gram-positive bacteria; however, the rest of the antibiotics were used for both isolates. The diameter of the zone of inhibition around each disc was measured to the nearest whole millimeter (mm) by using a ruler, and the isolate was classified as sensitive, intermediate, or resistant according to the standardized table supplied by CLSI, 2020^19^.

### Detection of extended spectrum *β*-lactamase (ESBL)

ESBL-producing *Escherichia coli and Klebsiella* spp*.* were first screened for ESBL production by the phenotypic method and then confirmed by the phenotypic confirmatory test as per CLSI guidelines 2020^19^.

#### Phenotypic screening for ESBL production

The ESBL screening test was performed by the standard disk diffusion method using ceftazidime (30 µg), cefotaxime (30 µg), and ceftriaxone (30 µg) (Oxoid, UK). More than one antibiotic disc was used for screening to improve the sensitivity of ESBL detection^19^. The three antibiotic discs were placed on Muller–Hinton agar and incubated at 37 °C for 18–24 h. These breakpoints for suspicion of ESBL production were: ≤ 25 mm for ceftriaxone (30 g), ≤ 22 mm for ceftazidime (30 g), and ≤ 27 mm for cefotaxime (30 g)^19^.

#### Phenotypic confirmation of ESBL producers

Confirmation of suspected ESBL producers was done by using the double-disk synergy (DDS) method. On Muller Hinton agar, an Amoxicillin/clavulanic acid (20/10 g) disc was placed in the center of the plate, and ceftazidime (30 g) and cefotaxime (30 g) discs were placed 15 mm apart, center to center. The plate was incubated at 37 °C for 18–24 h. A ≥ 5 mm increase in the diameter of the zone of inhibition for either of the cephalosporin-clavulanate disk combinations versus the zone diameter of the respective cephalosporin disk was considered positive, and the isolate was interpreted as an ESBL producer as recommended by CLSI guidelines 2020^19^.

#### Data processing and analysis

Data were entered using EpiData version 3.1 and exported to SPSS version 25 software. Descriptive statistics were used to summarize socio-demographic data, bacterial profile and susceptibility patterns of isolates. Bivariate logistic regression was employed to look for associations between the outcome variable and each independent variable; and those variables significant at a *P* value of less than 0.25 in the bivariate regression were then selected for the multivariate analysis model. The corresponding variables with a *P* value ≤ 0.05 at a 95% confidence interval were then considered statistically significant.

### Quality assurance

The legibility of the filled questionnaire and any labeling errors were confirmed immediately. Laboratory analyses were carried out using standard operating procedures. Culture media were tested for sterility and performance by incubating 5% of the batch at 35–37 °C overnight and observing the media for microbial growth. Those media which showed growth was discarded and replaced by a new sterile batch. Standard reference strains of *Staphylococcus aureus* (ATCC25923), *E. coli* (ATCC25922) and *Pseudomonas aeruginosa* (ATCC27853) were used during culture and antimicrobial susceptibility testing. *Escherichia coli* ATCC 25922 was used as an ESBL-negative whereas *K. pneumoniae* ATCC 700603 was used as an ESBL-positive reference strain^19^.

### Ethical consideration

The study protocol was evaluated and approved by the Institutional Review Board of Jimma University (Ref.: IRB000144/2020), and ethical clearance was obtained. The support letter was obtained from Jimma University School of Medical Laboratory Science and then submitted to JMC, gynecology ward and all methods were performed in accordance with relevant guidelines and regulations. After adequately explaining the objectives and purpose of the study, written informed consent was obtained from all the study subjects and/ or assent were obtained from study subject less than 18 years and/or guardians before data and sample collection. All data obtained in the course of the study was kept confidential. Positive cases were referred to the attending clinician as soon as possible for their better management.

### Ethics approval and consent to participate

Ethical permission for this study was approved by Jimma University, Research and Ethics Committee of the School of Medical Laboratory. All participants were voluntary and each supplied informed consent.

## Result

### Socio-demographic characteristics

A total of 386 patients admitted with gynecological cases were included in the present study. The mean age of the study participants was 31.9 ± 11.5 years, with an age range of 15–70 years. About 153 (39.6%) of study participants were in the age range of 25–34 years, and the majority were married (315, or 81.6%), high school (1–8; 35.2%) in their educational status, and urban dwellers (50.5%) in their residence. Based on their parity, the majority of 61.7% of the study participants were multiparous. Approximately 50.5% and 28% of the study participants had a history of catheterization and UTI, respectively (Table 1).

**Table 1:** Background characteristics of the gynecological cases (n = 386) in Jimma University Medical Centre, Ethiopia.

Variables	Category	Frequency	Percent (%)
Age (years)	15–24	114	29.5
	25–34	153	39.6
	35–44	67	17.4
	≥ 45	52	13.5
Occupation	Merchant	53	13.7
	Student	51	13.2
	House wife	220	57
	Employed	39	10.1
	Others	23	6
Educational status	No formal education	83	21.5
	1–8	136	35.2
	9–12	126	32.7
	College and above	41	10.6
Parity	Nullipara	83	21.5
	Primipara	65	16.8
	Multipara	238	61.7
Marital status	Single	53	13.7
	Divorced	18	4.7
	Married	315	81.6
History of UTI	Yes	108	28
	No	278	72
History of catheterization	Yes	195	50.5
	No	191	49.5

*ETB* Ethiopian birr, *UTI* urinary tract infection.

### The prevalence of UTIs and the types of bacteria isolated

**Figure 1:**
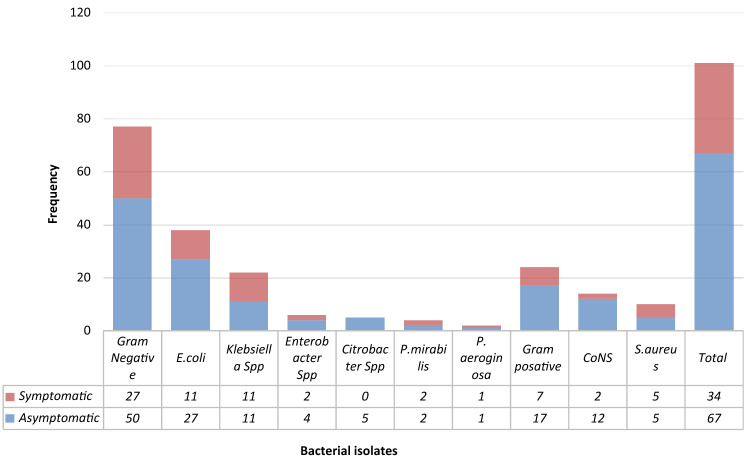
Frequency of bacterial uropathogens of symptomatic and asymptomatic UTI among gynecological cases admitted at Jimma University Medical Centre, Ethiopia.

Of the 386 urine specimens analyzed, 98 had significant bacteriuria, with an overall prevalence of 25.4%. A total of 101 bacterial isolates (bacterial species belonging to seven genera) were identified from the study participants. The prevalence of bacteriuria among patients with gynecological cases who had undergone gynecologic surgery was 73 (31.12%). Of these, 15 (20.5%) underwent emergency surgery and 58 (79.5%) underwent elective surgery. Among patients who had undergone non-surgical procedures the prevalence was 25 (16.4%) and 32 (34.4%) of the isolates were from symptomatic cases. The prevalence of asymptomatic bacteriuria was 66 (22.5%). Of the total 101 isolates, the majority (77, or 76.2%) were gram-negative organisms, while 24 (23.8%) were gram-positive bacteria. *Escherichia coli* was found to be the most frequent isolate 38 (49.4% of the gram-negatives, 37.6% of all isolates), followed by *Klebsiella* spp. 22 (21.8%) and CoNS 14 (13.9%). The other isolated organisms were *S.* *aureus* 10 (9.9%), *Enterobacter* spp. 6 (5.9%), *Citrobacter* spp. 5 (4.9%), *P. mirabilis* 4 (4%), and *P. aeroginosa* 2(2%). Of all, mixed pathogens were isolated from 3 (3.1%) patients (Fig. 1).

### Associated risk factors of UTI

Twelve independent variables were considered during the bivariate analysis of risk factors for significant bacteriuria. In multivariate analysis, histories of UTI (AOR = 1.977, 95% CI 1.06, 3.68, *P* = 0.032) and catheterization (AOR = 2.38, 95% CI 1.28, 4.45, *P* = 0.006) were found to have a statistically significant association with significant bacteriuria (Table 2).

**Table 2:** Bivariate and Multivariate Logistic Regression analyses of factors associated with significant bacteriuria among gynecological cases (n = 386) admitted at Jimma University Medical Centre, Ethiopia.

Variables	Category	Culture status		COR (95% CI)	AOR (95% CI)	*P* value
		SB (%)	NSB (%)			
Age in years	15–24	22 (19.3)	92 (80.7)	1	1	
	25–34	41 (26.8)	112 (73.2)	1.53 (0.85–2.75)	0.91 (0.32–2.59)	0.857
	35–44	17 (25.4)	50 (74.6)	1.42 (0.69–2.9)	0.61 (0.19–1.98)	0.411
	≥ 45	18 (34.6)	34 (65.4)	2.21 (1.06–4.63)	1.32 (0.40–4.37)	0.645
Educational level	No formal education	31 (37.3)	52 (62.7)	1.44 (0.64–3.23)	0.45 (0.07–2.84)	0.399
	1–8	28 (20.6)	108 (79.4)	0.63 (0.28–1.38)	0.36 (0.06–2.05)	0.251
	9–12	27 (21.4)	99 (78.6)	0.66 (0.30–1.46)	0.73 (0.14–3.95)	0.719
	College & >	12 (29.3)	29 (70.7)	1	1	
Marital status	Single	9 (17)	44 (83)	1	1	
	Divorced	7 (38.9)	11 (61.1)	3.1 (0.95–10.21)	0.73 (0.07–7.64)	0.793
	Married	82 (26)	233 (74)	1.7 (0.81–3.68)	0.49 (0.07–3.25)	0.462
Parity	Nullipara	14 (16.9)	69 (83.1)	1	1	
	Primipara	15 (23.1)	50 (76.9)	1.48 (0.66–3.34)	1.26 (0.21–7.55)	0.798
	Multipara	69 (29)	169 (71)	2.01 (1.06–3.81)	0.95 (0.17–5.24)	0.953
History of UTI	Yes	42 (38.9)	66 (61.1)	2.52 (1.55–4.10)	1.98 (1.06–3.68)	0.032*
	No	56 (20.1)	222 (79.9)	1	1	
History of catheterization	Yes	61 (31.3)	134 (68.7)	1.89 (1.19–3.03)	2.38 (1.28–4.45)	0.006*
	No	37 (19.4)	154 (80.6)	1	1	
Menopausal status	Pre	82 (24.3)	255 (75.7)	1	1	
	Post	16 (32.7)	33 (67.3)	1.51 (0.79–2.88)	1.06 (0.23–4.98)	0.938
Pregnancy	Yes	28 (17.9)	128 (82.1)	0.50 (0.30–0.82)	1.07 (0.23–4.49)	0.929
	No	70 (30.4)	160 (69.6)	1	1	
Current symptom	Yes	32 (34.4)	61 (65.6)	1.80 (1.09–3.00)	1.49 (0.77–2.88)	0.232
	No	66 (22.5)	227 (77.5)	1	1	
Duration of catheter in situ	1–3	43 (28.7)	107 (71.3)	1	1	
	1–6	9 (40.90)	13 (59.1)	1.72 (0.69–4.33)	1.14 (0.36–3.63)	0.825
	> 7	15 (53.6)	13 (46.4)	2.87 (1.26–6.54)	1.15 (0.34–3.88)	0.823
Duration of hospital stay	1–3	41 (21.7)	148 (78.3)	1	1	
	1–6	26 (24.8)	79 (75.2)	1.19 (0.68–2.08)	0.66 (0.28–1.58)	0.354
	> 7	31 (33.7)	61 (66.3)	1.83 (1.06–3.19)	0.72 (0.29–1.75)	0.463
Nature of surgery	Emergency	15 (23.8	48 (76.2)	0.61 (0.31–1.18)	0.72 (0.24–2.15)	0.560
	Elective	58 (33.9)		1	1	

*SB* significant bacteriuria, *NSB* non-significant bacteriuria, *AOR* adjusted odds ratio, *COR* crude odds ratio, *1* reference group, *95% CI* 95% confidence interval, *UTI* urinary tract infection.

*Statistically significant at *P* < 0.05.

### Antimicrobial susceptibility pattern of bacterial uropathogens

#### Gram negative bacteria

In this study, gram-negative uropathogens showed high levels of resistance to 68 (88.3%) for ampicillin and 51 (66.2%) for tetracycline. However, all gram-negative bacterial isolates showed a relatively low level of resistance against nitrofurantoin 21 (27.3%), norfloxacin 18 (23.4%), ceftazidime 17 (22.1%), and meropenem 3 (3.9%) (Table 3).

**Table 3:** Antimicrobial susceptibility pattern of gram-negative bacteria isolated from urine culture of gynecological cases admitted at Jimma University Medical Centre, Ethiopia.

Isolates	Pattern	STX	F	AMC	CTX	CN	NOR	NA	C	AMP	CiP	CRO	CAZ	TET	MEM
*E. coli* (38)	S	15 (39.5)	31 (81.6)	14 (36.8)	23 (60.5)	29 (76.3)	29 (76.3)	19 (50)	25 (65.8)	7 (18.4)	17 (44.7)	27 (71.1)	30 (78.9)	11 (28.9)	38 (100)
	I		2 (5.3)	4 (10.5)	2 (5.3)		1 (2.6)	1 (2.6)	4 (10.5)		5 (13.2)				
	R	23 (60.5)	5 (13.1)	20 (52.6)	13 (34.2)	9 (23.7)	8 (21.1)	18 (47.4)	9 (23.7)	31 (81.6)	16 (42.1)	11 (28.9)	8 (21.1)	27 (71.1)	
*K. pneum-oniae* (21)	S	7 (33.3)	11 (52.4)	3 (14.3)	12 (57.1)	4 (19)	14 (66.6)	12 (57.2)	5 (23.8)		6 (28.6)	12 (57.1)	14 (66.7)	6 (28.6)	19 (90.5)
	I	1 (4.8)	2 (9.5)	2 (9.5)	1 (4.8)	2 (9.5)	1 (4.8)	2 (9.5)	3 (14.3)		7 (33.3)		2 (9.5)	2 (9.5)	
	R	13 (61.9)	8 (38.1)	16 (76.2)	8 (38.1)	15 (71.4)	6 (28.6)	7 (33.3)	13 (61.9)	21 (100)	8 (38.1)	9 (42.9)	5 (23.8)	13 (61.9)	2 (9.5)
*K.oxytoca* (1)	S								1 (100)						
	I														
	R	1 (100)	1 (100)	1 (100)	1 (100)	1 (100)	1 (100)	1 (100)		1 (100)	1 (100)	1 (100)	1 (100)	1 (100)	1 (100)
*P.mira- bilis* (4)	S		1 (25)	1 (25)	2 (50)	1 (25)	3 (75)	1 (25)	2 (50)			3 (75)	3 (75)	1 (25)	4 (100)
	I			2 (50)				1 (25)							
	R	4 (100)	3 (75)	1 (25)	2 (50)	3 (75)	1 (25)	2 (50)	2 (50)	4 (100)	4 (100)	1 (25)	1 (25)	3 (75)	
*Citroba- cter* spp (5)	S	4 (80)	5 (100)	2 (40)	4 (80)	3 (60)	2 (40)	3 (60)	2 (40)			4 (80)	4 (80)	2 (40)	5 (100)
	I			2 (40)			2 (40)				3 (60)			1 (20)	
	R	1 (20)		1 (20)	1 (20)	2 (40)	1 (20)	2 (40)	3 (60)	5 (100)	2 (40)	1 (20)	1 (20)	2 (40)	
*P.aerogi*-*nos*a (2)	S			1 (50)		1 (50)	2 (100)		1 (50)		2 (100)		2 (100)	1 (50)	2 (100)
	I	1 (50)			1 (50)				1 (50)						
	R	1 (50)	2 (100)	1 (50)	1 (50)	1 (50)		2 (100)		2 (100)		2 (100)		1 (50)	
*Enteroba*-*cter* spp (6)	S	2 (33.3)	3 (50)	3 (50)	3 (50)	2 (33.3)	5 (83.3)	3 (50)	4 (66.7)	2 (33.3)	4 (66.7)	3 (50)	5 (83.3)	2 (33.3)	6 (100)
	I	1 (16.7)	1 (16.7)		2 (33.3)			1 (16.7)			2 (33.3)				
	R	3 (50)	2 (33.3)	3 (50)	1 (16.7)	4 (66.7)	1 (16.7)	2 (33.3)	2 (33.3)	4 (66.7)		3 (50)	1 (16.7)	4 (66.7)	
Total (77)	S	28 (36.4)	51 (66.2)	24 (31.2)	44 (58.4)	40 (51.9)	55 (71.4)	38 (49.3)	40 (51.9)	9 (11.7)	29 (37.7)	49 (63.6)	58 (75.3)	23 (29.9)	74 (96.1)
	I	3 (3.9)	5 (6.5)	10 (13)	6 (7.8)	2 (2.6)	4 (5.2)	5 (6.5)	8 (10.4)		17 (22)		2 (2.6)	3 (3.9)	
	R	46 (59.7)	21 (27.3)	43 (55.8)	27 (35.1)	35 (45.5)	18 (23.4)	34 (44.2)	29 (37.7)	68 (88.3)	31 (40.3)	28 (36.4)	17 (22.1)	51 (66.2)	3 (3.9)

*AMP* Ampicillin, *TET* Tetracycline, *CiP* Ciprofloxacin, *F* Nitrofurantoin, *P* Penicillin, *CRO* Ceftriaxone, *STX* Trimethoprim- Sulfamethoxazole, *AMC* Amoxicillin- clavulanic acid, *CN* Gentamycin, *NOR* Norfloxacin, *C* Chloramphenicol, *MEM* Meropenem, *CTX* Cefotaxime, *CAZ* Ceftazidime.

#### Gram-positive bacteria

Out of the tested antibiotics, gram-positive bacterial isolates showed a high level of resistance to penicillin (91.6%) and trimethoprim-sulfamethoxazole (66.7%). On the other hand, all gram-positive isolates showed full sensitivity to 100.0% nitrofurantoin. Moreover, gram-positive isolates showed sensitivity towards clindamycin 79.2%, ciprofloxacin 75.0%, norfloxacin 87.5%, and amoxicillin-clavulanic acid 91.6% (Table 4).

**Table 4:** Antimicrobial susceptibility pattern of gram-positive bacteria isolated from urine culture of gynecological cases admitted at Jimma University Medical Centre, Ethiopia.

Isolates	Pattern	STX	F	AMC	CN	NOR	C	AMP	CiP	CRO	E	P	DA
CoNS (14)	S	3 (21.3)	14 (100)	12 (85.7)	7 (50)	12 (85.7)	8 (57.1))	4 (28.6)	12 (85.7)	12 (85.7)	5 (35.7)		13 (92.9)
	I			1 (7.1)	3 (21.4)	1 (7.1)		1 (7.1)	1 (7.1)		4 (28.6)		
	R	11 (78.7)		1 (7.1)	4 (28.6)	1 (7.1)	6 (42.9)	9 (64.3)	1 (7.1)	2 (14.3)	5 (35.7)	14 (100)	1 (7.1)
*S. aureus* (10)	S	5 (50)	10 (100)	10 (100)	7 (70)	9 (90)	6 (60)	3 (30)	6 (60)	7 (70)	6 (60)	2 (20)	6 (60)
	I				1 (10)	1 (10)		2 (20)	1 (10)				2 (20)
	R	5 (50)			2 (20)		4 (40)	5 (50)	3 (30)	3 (30)	4 (40)	8 (80)	2 (20)
Total (24)	S	8 (33.3)	24 (100)	22 (91.6)	14 (58.3)	21 (87.5)	14 (58.3)	7 (29.2)	18 (75)	19 (79.2)	15 (62.5)	2 (8.3)	19 (79.2)
	I			1 (4.2)	4 (16.7)	2 (8.3)		3 (12.5)	2 (8.3)				2 (8.3)
	R	16 (66.7)		1 (4.2)	6 (25)	1 (4.2)	10 (41.7)	14 (58.3)	4 (16.7)	5 (20.8)	9 (37.5)	22 (91.6)	3 (12.5)

*AMP* Ampicillin, *TE* Tetracycline, *CiP* Ciprofloxacin, *E* Erythromycin, *DA* Clindamycin, *F* Nitrofurantoin, *P* Penicillin, *CRO* Ceftriaxone, *STX* trimethoprim-sulfamethoxazole, *AMC* Amoxicillin-clavulanic acid, *CN* Gentamycin, *NOR* norfloxacin, *C* chloramphenic.

### Multidrug resistance pattern of the isolates

Among the total isolates (n = 101), overall, 96 (95.0%) bacterial isolates were resistant to at least one antimicrobial agent, whereas 87 (86.1%) isolates were resistant to ≥ 2 antimicrobials. Multidrug resistance (defined as non-susceptible to ≥ 1 agent in ≥ 3 antimicrobial categories)^20^ was seen in 80 (79.2%) of all isolated bacterial uropathogens. 62 (80.5%) of gram-negative and 18 (75%) of gram-positive bacteria showed multidrug resistance for the tested antimicrobial drugs. *Escherichia coli* and *K. pneumoniae* were found to be highly resistant to most of the antibiotics tested (Table 5).

**Table 5:** Level of Antimicrobial Resistance of isolated bacterial uropathogens among gynecological cases admitted at Jimma University Medical Centre, Ethiopia.

Bacterial isolates	Total	Antimicrobial resistance pattern							ESBL producers
		R0	R1	R2	R3	R4	≥ R5	MDR	
Gram negatives	77 (69.3)	4 (5.2)	5 (6.5)	9 (11.7)	9 (11.7)	4 (5.2)	46 (59.7)	62 (80.5)	16
*E. coli*	38 (37.6)	3 (7.9)	3 (7.9)	6 (15.8)	6 (15.8)	1 (2.6)	19 (50)	27 (71.1)	10
*K. pneumoniae*	21 (20.8)	0	0	2 (9.5)	2 (9.5)	1 (4.8)	16 (76.2)	20 (95.2)	6
*K. oxytoca*	1 (1)	0	0	0	0	0	1 (100)	1 (100)	0
*P. mirabilis*	4 (4)	0	0	0	0	0	4 (100)	4 (100)	0
*Enterobacter* spp.	6 (5.9)	1 (16.7)	1 (16.7)	0	0	0	4 (66.7)	4 (66.7)	0
*P. aeruginosa*	2 (2)	0	0	0	0	1 (50)	1 (50)	2 (100)	0
*Citrobacter* spp*.*	5 (5)	0	1 (20)	1 (20)	1 (20)	1 (20)	1 (20)	4 (80)	0
Gram positives	24 (23.8)	1 (4.2)	4 (16.7)	4 (16.7)	5 (20.8)	5 (20.8)	5 (20.8)	18 (75)	NT
*CoNS*	14 (13.9)	0	2 (14.3)	4 (28.6)	4 (28.6)	1 (7.1)	3 (21.4)	10 (71.4)	NT
*S. aureus*	10 (9.8)	1 (10)	2 (20)	0	1 (10)	4 (40)	2 (20)	8 (80)	NT
Total	101 (100)	5 (5)	9 (8.9)	12 (11.9)	16 (15.8)	11 (10.9)	48 (47.5)	80 (79. 2)^P^	16 (26.7)

Non-susceptible to > 1 agent in > 3 antimicrobial categories. ^P^ Percent is computed from total number of isolates, based on which MDR definition is applied.

*R0* no antibiotic resistance, *R1* resistance to one, *R2* resistance to two, *R3* resistance to three, *R4* resistance to four, ≥ *R5* resistance to five and more drugs, *MDR* multidrug resistant.

### ESBL-producing uropathogens

The isolates were *E. coli* 38(49.3%), *K. pneumoniae* 21 (27.3%), *Enterobacter* spp. 6(7.8%), *P. mirabilis* 4(5.2%), *Citrobacter* spp. 5(6.5%), *P. aeruginosa* 2(2.6%), and *K. oxytoca* 1(1.3%). In this study, *E. coli* and *Klebsiella* spp. were the most frequently isolated bacteria, and the methods were validated for them. Of the 60 g-negative isolates of *E. coli* and *Klebsiella* spp., 16 (26.7%) were positive for ESBL production, with 10 (26.3%) of the *E. coli* isolates and 6 (27.3%) of *Klebsiella* spp. All ESBL-producing isolates showed 100% resistance to ceftriaxone, cefotaxime, and ceftazidime, 87.5% of drugs. However, all the ESBL-producing uropathogens were sensitive to meropenem at 100% (Table 5).

## Discussion

In this study, the overall prevalence of significant bacteriuria among admitted gynecological cases was found to be 25.4%, which was in agreement with the findings of the previous studies conducted in India 24%^21^, Nigeria, 26.2%^22^ and USA 22.4%^23^. However, our finding was higher than other studies reported from Norway, 16.3%^24^ and USA, 11.3%^25^. But, our finding was lower than the reports from Ethiopia, 58.1%^26^ and Nigeria, 76.1%^27^. This inconsistency in prevalence might be due to differences in the sample size, methods employed, standard of personal hygiene, predisposing factors, and study population. Furthermore, the prevalence of uropathogenic bacteria observed in our study is still high compared to those reported in developed countries 6%^28^ and this might be due to a difference in the level of health-care development^29^.

In this study, gram-negative bacteria isolates were more prevalent with 77 (76.2%) than gram-positive bacteria isolates with 24 (23.8%). This could be due to the presence of a unique structure in gram-negative bacteria that helps with the attachment to the uroepithelial cells and prevents bacteria from being washed away by urine, allowing for multiplication and tissue invasion^30^.

E. coli was the most frequent etiological agent of UTI, accounting for up to 37.6% of isolated cases. This finding is in agreement with the findings from Ethiopia, 35.7%^31^, Nigeria, 34.2%^32^ and India, 37.3%^33^. The possible explanation for this high isolation rate of *E. coli* in the present finding could be due to the significant abundance of *E. coli* in the rectal area, which in turn via contamination ascends through genitalia to the urinary tract and causes UTI and it could also be due to *E. coli* having various enhanced virulence factors specific for colonization and invasion of the urinary epithelium, such as P-fimbriae and S-fimbriae adherence factors which mediate the attachment of *E. coli* to viginal and uroepithelial cells^34^.

Patients with gynecological cases who underwent gynecologic surgery had 72 (30.9%) positive cultures, whereas 26 (17%) of the 153 patients who underwent non-surgical procedures had significant bacteriuria, indicating that patients with gynecological cases who underwent gynecologic surgery were more affected than those who did not. This might be due to the close proximity of the rectum to the operating field and the use of urinary catheters, which aid in emptying the bladder during the procedure, which has a strong association with the development of UTI^35^.

This study's findings also show that a history of UTI has a strong association with significant bacteriuria, as shown in Table 2. Similar finding were reported from Ethiopia^26,36^. This is possibly due to the presence of resistant bacterial strains from those who had a previous history of UTI after poor diagnosis and ineffective treatment.

The prevalence of uropathogenic bacteria in admitted gynecological cases with a history of catheterization was also significantly higher than those without a history of catheterization, which is almost similar to other reports where catheterization is the most important risk factor for the development of catheter associated bacteriuria in Northern America^37^ and Ethiopia^26^ this is possibly due to catheterization, which could cause urethral mucosa injury when staying for a long time and might induce hematogenous bacterial spread in the urinary tract, the formation of biofilm by gram negative bacteria, and contamination during the insertion of catheters^38,39^.

Symptoms were not associated with the significant bacteriuria in this study. From 93 patients with gynecological pathologies who complain of having symptoms that suggest symptomatic urinary tract infection, only 32/63 (34.4%) were found to have culture confirmed urinary tract infection. Similar findings were also reported in Ethiopia^26^. Symptomatic patients whose urine cultures did not show significant growth might be due to several different microorganisms that can cause UTIs, including protozoan parasites, fungi, and viruses, even though bacteria are the major causative organisms^40^.

In this study, 26.7% of the gram-negative (*E. coli* and *Klebsiella* spp.) isolates were found to be ESBL producers. ESBL production of *Klebsiella* isolates was 27.3%, which is slightly higher than previous findings in Jimma, Ethiopia of 23.5%^41^. The rise in the prevalence of ESBL-producing uropathogens in the current study might be due to the fact that our study participants were all hospitalized, since hospitalization was identified as the strongest independent risk factor for ESBL^42^*.*

In this study, among gram-negative bacteria, the highest resistance was shown to ampicillin 88.3%, followed by tetracycline 66.2%. The reason may be due to the continuous use of these drugs for many years. High level of resistance to beta-lactam ring containing antibiotics could happen because of the presence of extended spectrum β-lactamase in these strains. On the other hand, about 74/77 (96.1%) of isolates were sensitive to meropenem. This might be due to the unavailability of this drug in the area. In addition, lower resistance (higher rate of sensitivity) was observed against 51/77 (66.2%) nitrofurantoin, 55 (71.4%) norfloxacin and 58/77 (75.3%) ceftazidime. The possible justification for such low-level resistance might be attributable to the infrequent prescription of these drugs. Hence, they could be considered as alternative options in the treatment of UTI.

Among the gram-negatives, the predominant isolate was *E. coli*, which is sensitive to nitrofurantoin 81.6%, ceftriaxone 78.9% and norfloxacin 76.3%. This was consistent with findings in Addis Ababa, Ethiopia^43^, while high resistance was shown to ampicillin (31; 81.6%) and tetracycline (27; 71.1%), which is in line with studies from Dire-Dawa, Ethiopia^44^ and Gondor, Ethiopia^45^. In this study, *Klebsiella* spp*. showed a* high rate of sensitivity to norfloxacin (14; 66.7%) and ceftriaxone (14; 66.7%), which is in line with a study done at Gondor, Ethiopia^45^. Also, the isolates showed high resistance to ampicillin 22 (100%) and amoxicillin-clavulanic acid 17 (77.3%), which is similar to the study done by Belete et al.^46^. The possible explanation of ampicillin and amoxicillin-clavulanic acid resistance might be due to their extensive use in health facilities, as this may augment the selection of uropathogens harboring the β-lactamase enzyme, which can hydrolyze penicillin.

Gram-positive bacteria were relatively resistant to penicillin (91.6%) and trimethoprim-sulfamethoxazole (66.7%). This might be due to the easy availability and indiscriminate use of these drugs, which could lead to an increase in resistance. On the contrary, all tested gram-positive isolates showed sensitivity to nitrofurantoin 100.0%. The reason for the effectiveness of this drug might be due to its narrow range of clinical indications, which results in less usage.

According to the international standard for the definition of drug resistance^20^, multidrug resistance (non-susceptible to ≥ 1 agent in ≥ 3 antimicrobial categories) was observed in 79.2% of the total isolated bacterial uropathogens, which was comparable with the finding from North East Ethiopia of 80.4%^46^. However, our finding was lower than previously reported MDR prevalence in Ethiopia (95–100%)^44,45^ and Kenya 96.0%^47^. In contrast, the result of this study was higher than studies from other documented results in Ethiopia, 74.0%^48^. The possible reason for this rise in MDR might be the repeated, inappropriate, and incorrect use of antimicrobial agents in empirical treatment, which in turn raises the prevalence of resistant microorganisms in the community^44^. It could also be due to multiple resistant genes that can develop on the mobile genetic elements^49^ and plasmids bearing genes-encoding ESBLs, which frequently also carry genes encoding resistance to other antimicrobial agents^14^.

All ESBL-producing isolates showed 100.0% sensitivity to meropenem. Therefore, carbapenems are considered the drugs of choice in the treatment of severe infections caused by ESBL-producing bacteria^50^. All of the ESBL positive isolates showed a high level of resistance; 100.0% to ampicillin, sulphamethoxazole-trimethoprim, ceftriaxone, cefotaxime, and 87.5% to ceftazidime. The treatment of choice for ESBL-producing bacteria is very limited^14^. Such findings indicate that the use of these antibiotics for the treatment of urinary tract infections caused by ESBL-positive strains may not produce the desired effect and may result in a significant amount of treatment failure. Conversely, ESBL-positive strains can respond better to carbapenem drugs such as meropenem, which could be a better treatment option.

Limitations of the Study was, it focused on admitted patients until the time of sample collection, before discharge and didn’t investigated infections that may have developed after the patients were discharged. In addition, molecular techniques for ESBL confirmation and pathogen identification were not carried out.

## Conclusions

The overall prevalence of UTI among admitted gynecological cases was 25.4%. *Escherichia coli* was the most dominant pathogen, which accounted for 37.6% of the isolates, followed by *Klebsiella* spp*.* The history of UTI and history of catheterization had a statistically significant association with the uropathogenic bacterial isolates. This study also showed that Nitrofurantoin and Norfloxacin were the drugs of choice for both gram-negative and gram-positive bacteria, whereas ampicillin and trimethoprim-sulfamethoxazole were less effective for the management of UTI among our study participants. While meropenem and clindamycin were found effective against gram-negative and positive bacteria isolated, respectively, a significant number of ESBL producers (26.7%) and an alarmingly high multi-drug resistance have been shown in most of the bacterial isolates (79.2%). Therefore, screening admitted gynecological patients, especially for those who have history of catheterization and UTI, by urine culture and antimicrobial susceptibility testing. Furthermore, antibiotic stewardship program should start at the hospital to reduce the observed antibiotic resistance and prevent further complications.

## Data Availability

The datasets used and/or analyzed during the current study are available from the corresponding author on reasonable request.

## Abbreviations

AOR: Adjusted odds ratio
ATCC: American Type Culture Collection
BAP: Blood agar plate
CFU: Colony forming unite
CLSI: Clinical Laboratory Standard Institute
CoNS: Coagulase negative staphylococci
COR: Crude odds ratio
DDS: Double-disk synergy
ESBL: Extended spectrum B-lactamase
JUMC: Jimma University Medical Center
KIA: Kligler’s iron agar
MSU: Mid-stream urine
QC: Quality control
SIM: Sulphur indole motility
SOP: Standard operating procedure
SPSS: Statistical package for social science
UK: United Kingdom
UTI: Urinary tract infection
WHO: World Health Organization
